# Serotonin-immunoreactive neurons in the ventral nerve cord of Remipedia (Crustacea): support for a sister group relationship of Remipedia and Hexapoda?

**DOI:** 10.1186/1471-2148-13-119

**Published:** 2013-06-10

**Authors:** Torben Stemme, Thomas M Iliffe, Björn M von Reumont, Stefan Koenemann, Steffen Harzsch, Gerd Bicker

**Affiliations:** 1 Division of Cell Biology, University of Veterinary Medicine Hannover, Bischhofsholer Damm 15, 30173, Hannover, Germany; 2 Department of Marine Biology, Texas A&M University at Galveston, 200 Seawolf Parkway, Galveston, TX 77553, USA; 3 Department of Life Sciences, Natural History Museum London, Cromwell, Road SW7 5BD, United Kingdom; 4 Montessori Bildungshaus Hannover, Bonner Straße 10, 30173, Hannover, Germany; 5 Department of Cytology and Evolutionary Biology, Institute of Zoology, Ernst-Moritz-Arndt-University of Greifswald, Soldmannstraße 23, 17487, Greifswald, Germany

**Keywords:** Euarthropoda, Homology, Comparative neuroanatomy, Ground pattern, Phylogeny, Immunocytochemistry, *Speleonectes*, *Godzilliognomus*, *Cryptocorynetes*

## Abstract

**Background:**

Remipedia were initially seen as a primitive taxon within Pancrustacea based on characters considered ancestral, such as the homonomously segmented trunk. Meanwhile, several morphological and molecular studies proposed a more derived position of Remipedia within Pancrustacea, including a sister group relationship to Hexapoda. Because of these conflicting hypotheses, fresh data are crucial to contribute new insights into euarthropod phylogeny. The architecture of individually identifiable serotonin-immunoreactive neurons has successfully been used for phylogenetic considerations in Euarthropoda. Here, we identified neurons in three species of Remipedia with an antiserum against serotonin and compared our findings to reconstructed ground patterns in other euarthropod taxa. Additionally, we traced neurite connectivity and neuropil outlines using antisera against acetylated α-tubulin and synapsin.

**Results:**

The ventral nerve cord of Remipedia displays a typical rope-ladder-like arrangement of separate metameric ganglia linked by paired longitudinally projecting connectives. The peripheral projections comprise an intersegmental nerve, consisting of two branches that fuse shortly after exiting the connectives, and the segmental anterior and posterior nerve. The distribution and morphology of serotonin-immunoreactive interneurons in the trunk segments is highly conserved within the remipede species we analyzed, which allows for the reconstruction of a ground pattern: two posterior and one anterior pair of serotonin-immunoreactive neurons that possess a single contralateral projection. Additionally, three pairs of immunoreactive neurons are found in the medial part of each hemiganglion. In one species (*Cryptocorynetes haptodiscus*), the anterior pair of immunoreactive neurons is missing.

**Conclusions:**

The anatomy of the remipede ventral nerve cord with its separate metameric ganglia mirrors the external morphology of the animal’s trunk. The rope-ladder-like structure and principal architecture of the segmental ganglia in Remipedia corresponds closely to that of other Euarthropoda. A comparison of the serotonin-immunoreactive cell arrangement of Remipedia to reconstructed ground patterns of major euarthropod taxa supports a homology of the anterior and posterior neurons in Pancrustacea. These neurons in Remipedia possess unbranched projections across the midline, pointing towards similarities to the hexapod pattern. Our findings are in line with a growing number of phylogenetic investigations proposing Remipedia to be a rather derived crustacean lineage that perhaps has close affinities to Hexapoda.

## Background

A variety of independent molecular and morphological studies strongly supports a close relationship of Hexapoda and Crustacea (e.g., [1-3]), known as Tetraconata or Pancrustacea [4-6]. However, which crustacean taxon represents the possible sister group of Hexapoda is still under debate. Quite a number of crustacean taxa were suggested to be closest relatives to Hexapoda in different kinds of analyses (see review [7]), for example all Crustacea, or subgroups such as Cephalocarida, Copepoda, Branchiopoda, Malacostraca, Remipedia or Xenocarida (Remipedia and Cephalocarida; [8]). Most of these predominantly molecular studies did not provide a comprehensive crustacean taxon sampling, which resulted in the lack of important groups, e.g., the Remipedia [9].

**Figure 1:**
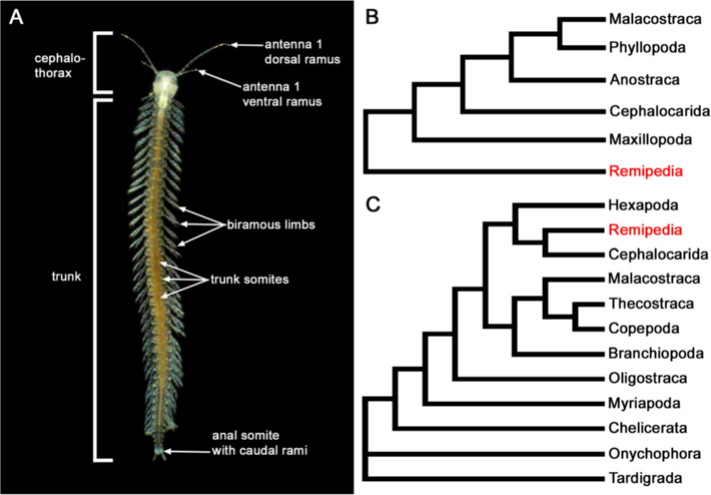
**External morphology of Remipedia and two hypotheses of their phylogenetic position.** (**A**) Remipedia are composed of a cephalothorax and a homonomously segmented trunk consisting of numerous trunk somites equipped with biramous limbs (Photograph of *Speleonectes tanumekes* adapted from [22,66]; photograph courtesy of J. van der Ham). (**B**) Because Remipedia have many characters that are considered plesiomorphic, for example the metameric trunk, they were seen as the basal sister group to all other Crustacea in early morphological studies (adapted from [13]). (**C**) In the last decade, several morphological and molecular analyses suggested Remipedia to be a more derived lineage within Pancrustacea (adapted from [8]).

Remipedia are cave-dwelling, homonomously segmented, pale and eyeless Crustacea (Figure 1A) that play a key role in understanding pancrustacean phylogeny. Almost all possible phylogenetic affinities were proposed for this taxon, e.g., sister group relationships to all other Crustacea, or to subgroups such as Cirripedia, Ostracoda, Cephalocarida, “maxillopodan” taxa, or to Hexapoda (reviewed in [7]). Because Remipedia have many morphological characters that were considered plesiomorphic, for example the homonomously segmented trunk, paddle-like appendages and a cephalic shield, they were initially seen as the sister group to all other Crustacea or Pancrustacea in early morphological studies (e.g., [10-13]; Figure 1B). In contrast, newer comparisons of molecular sequence information together with studies of the brain anatomy, ovary structure and development changed this view and proposed Remipedia to be closely related to more derived pancrustacean lineages such as Malacostraca or Hexapoda (Figure 1C) [8,9,14-22]. In several phylogenetic analyses, Remipedia often clustered together with Cephalocarida [8,18,19,23-27]. In a study by Regier et al., this clade has been named “Xenocarida”, a group that together with Hexapoda forms the “Miracrustacea” [8]. However, the clade “Xenocarida” has not been recovered by a recent analysis combining morphology and molecular sequence information, which instead favors a sister group relationship of Remipedia and Hexapoda without Cephalocarida [28]. Similar to the situation in Remipedia, the phylogenetic affinities of other crustacean groups, for example that of Cephalocarida and Branchiopoda, are far from clear [7,9]. Because morphological comparisons and molecular sequence analyses have often led to contradicting hypotheses, there is a need for additional information. Along these lines, the arrangement and neurochemical architecture of the nervous system has proven to contain a variety of valuable characters (e.g., [29-32]) that can help to unravel euarthropod phylogeny. The immunocytochemical localization of neuroactive compounds provides information on the anatomy and certain biochemical pathways expressed in a particular neuron. This approach offers the rather unique possibility of establishing homologies at the level of single cells between distantly related species.

Serotonin-immunoreactive (5HT-ir) neurons are suitable for phylogenetic investigations within Euarthropoda for several reasons [33]. The number of 5HT-ir neurons is small, facilitating individual identification of cells and their characteristic neurite morphology. Additionally, the serotonin-immunoreactivity (5HT-IR) has been investigated in a wide range of euarthropod species allowing comparisons over a wide range of taxa (Chelicerata and Myriapoda: e.g., [34-36]; Crustacea: e.g., [33,37-43]; Hexapoda: e.g., [44-47]). Furthermore, serotonergic neurons have not only been analyzed in Euarthropoda, but also in other invertebrates and vertebrates, which led to the suggestion that the serotonergic system is highly conserved in evolution [48].

While the general morphology and the 5HT-IR of the remipede brain have already been analyzed [14,15,22], here, we describe the neuroanatomy of the ventral nerve cord focusing on the distribution and morphology of 5HT-ir neurons in three species of Remipedia. Our comparisons to already studied euarthropod taxa add new insights to the phylogenetic position of Remipedia and the crustacean sister group of Hexapoda.

## Results

### General neuroanatomy of the ventral nerve cord of Remipedia

**Figure 2:**
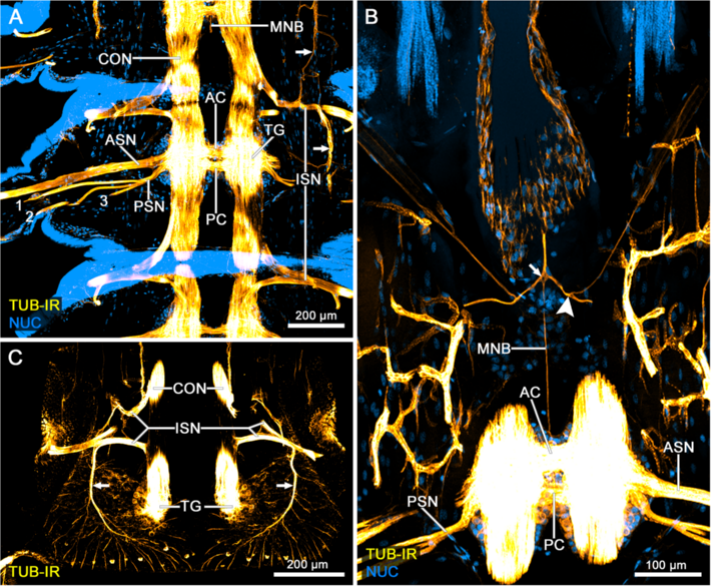
**General morphology of the ventral nervous system of *Speleonectes tulumensis*.** (**A-C**) Dorsal view on confocal laser-scans of horizontal vibratome sections (50 μm) through the trunk of *Speleonectes tulumensis* labeled for acetylated α-tubulin-immunoreactivity (TUB-IR, yellow) and nuclear marker (NUC, blue). (**A**) Each trunk ganglion (TG) comprises an anterior and a posterior commissure (AC and PC) connecting both hemiganglia. Two segmental nerves (ASN and PSN) leave each hemiganglion laterally and project into the appendages. The posterior segmental nerve (PSN) splits into three branches (numbers 1–3). Furthermore, an intersegmental nerve (ISN) innervates the periphery and sends neurite bundles anteriorly and posteriorly (arrows) parallel to the connectives (CONs). These longitudinal CONs and a slender longitudinal median neurite bundle (MNB) connect the segmental ganglia of proximate segments. (**B**) Between the segmental ganglia, two branches leave the MNB posterolaterally (arrow) and split immediately into two branches (arrowhead), one of which projects anterolaterally. (**C**) One branch of the ISN grows posteriorly and ventrally (arrow) and splits in numerous radially distributed neurites. Abbreviations: numbers 1–3: neurite bundles originating from the posterior segmental nerve; AC: anterior commissure; ASN: anterior segmental nerve; CON: connective; ISN: intersegmental nerve; MNB; unpaired longitudinal median neurite bundle; NUC: nuclear marker; PSN: posterior segmental nerve; TG: trunk ganglion; TUB-IR: acetylated α-tubulin-immunoreactivity.

**Figure 3:**
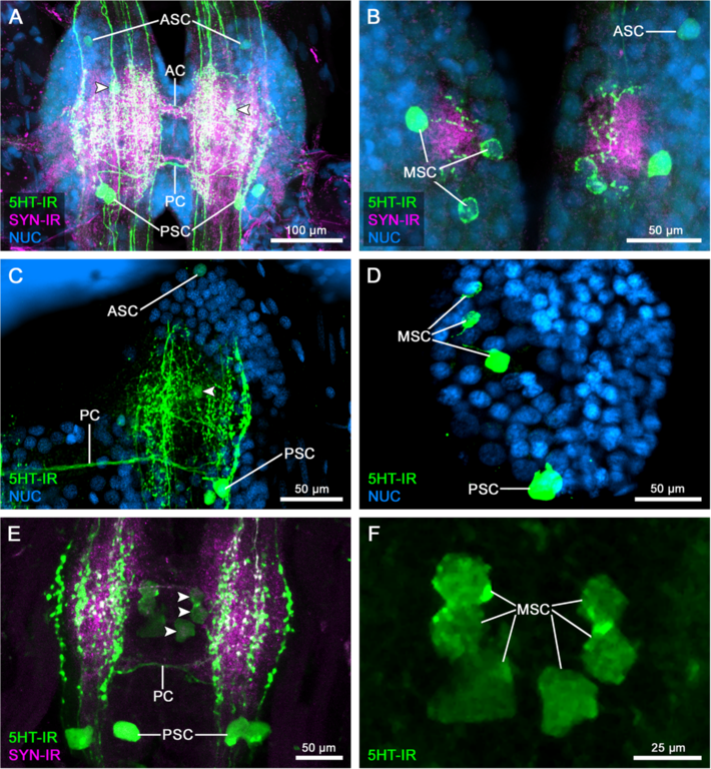
**Serotonin**-**immunoreactive neurons in the ventral nervous system of Remipedia.** Maximum projections of confocal laser-scans of trunk ganglia of *Godzilliognomus frondosus* (**A, B**), *Speleonectes tulumensis* (**C, D**), and *Cryptocorynetes haptodiscus* (**E,F**) labeled for serotonin-immunoreactivity (5HT-IR, green), synapsin-immunoreactivity (SYN-IR, magenta) and nuclear marker (NUC, blue). (**A**) One anterior pair of 5HT-ir cells (ASC) and two pairs of posterior 5HT-ir cells (PSC) are clearly visible. Additionally, at least one cell pair in a medial position (MSC) could be detected (arrowheads). (**B**) Higher magnification of a more ventral plane of the same ganglion shown in (**A**). The absence of the strongly labeled neuropil reveals in total three pairs of medial 5HT-ir cells (MSC). In this example, these neurons do not possess a bilateral symmetric arrangement (compare to F). (**C**) The right hemiganglion of a trunk segment in *Speleonectes tulumensis* is shown. Two PSCs, one ASC and at least one MSC (arrowhead) are similarly distributed as in *Godzilliognomus frondosus*. (**D**) Higher magnification of the most ventral part of another right hemiganglion of *Speleonectes tulumensis*. Beside two PSCs, three neurons are distributed in the medial part of the hemiganglion (ASC out of focus). (**E**) In the trunk ganglion of *Cryptocorynetes haptodiscus*, two PSCs show strong 5HT-IR. Additionally, three weakly labeled cells are visible in the medial part (arrowheads). (**F**) Higher magnification of the MSCs shown in (**E**). In this example, these neurons are arranged nearly in bilateral symmetry (compare to **B**). Abbreviations: 5HT-IR: serotonin-immunoreactivity; AC: anterior commissure; ASC: anterior 5HT-ir cell; MSC: medial 5HT-ir cell; NUC: nuclear marker; PC: posterior commissure; PSC: posterior 5HT-ir cell; SYN-IR: synapsin-immunoreactivity.

The ventral nerve cord in the trunk segments of the investigated species, of Remipedia displays a rope-ladder-like arrangement of separate metameric ganglia (Figure 2A). Each segmental ganglion is clearly divided into a neuropil core and a surrounding soma cortex that forms a bulge ventrolaterally (Figure 3A). The neuropil of the two bilateral segmental hemiganglia are connected by an anterior and a posterior commissure (AC and PC) (Figures 2A and 3A). The trunk ganglia (TGs) are linked by a pair of longitudinal connectives (CONs) (Figure 2A). In addition, a slender unpaired longitudinal median neurite bundle (MNB) links the ganglia of the ventral nerve cord (Figure 2A, B). Two slender branches leave this neurite bundle in a posterolateral direction between the segmental ganglia (Figure 2B). After a short distance, these branches split again into two fibers, the thicker one growing laterally, the more slender one anterolaterally into the periphery (Figure 2B).

The innervation of the trunk segments is provided by the anterior and posterior segmental nerves (ASN and PSN) that exit each ganglion laterally. The thicker ASN originates at a level between the AC and PC, projecting posterolaterally straight into the appendages (Figure 2A, B). The PSN leaves the ganglion laterally of the PC parallel to the ASN. Subsequently, it splits into three branches, at least two of which run into the appendages (Figure 2A, B).

Additionally, two neurite bundles leave the CON. Both bundles eventually fuse, forming an intersegmental nerve (ISN) that extends further laterally into the periphery (Figure 2A, C). This nerve then divides several times forming a network of neurite bundles distally to the ventral nerve cord. One anterior and one posterior projecting neurite bundle can be distinguished (arrows in Figure 2A). The posterior projecting branch of the ISN extends ventrally and finally splits in numerous radially distributed fine neurite bundles (Figure 2C).

### Serotonin-immunoreactive neurons in the ventral nerve cord

In all remipede species examined here, the 5HT-ir neurons of the ventral ganglia are arranged in a stereotyped pattern. Based on their soma position within each ganglion, three groups can be distinguished. In *Speleonectes tulumensis* and *Godzilliognomus frondosus*, we detected six pairs of 5HT-ir neurons (Figure 3A-D). These individually identifiable neurons are arranged in two posterior, three medial, and one anterior pair of immunoreactive neurons (PSC, MSC, and ASC, respectively). All these neurons are found in a ventral position of the nervous system. In general, the distribution and projections of 5HT-ir neurons appear similar in all three investigated species. One conspicuous modification is observed in *Cryptocorynetes haptodiscus*, namely the absence of the ASC (Figure 3E, F).

**Figure 4:**
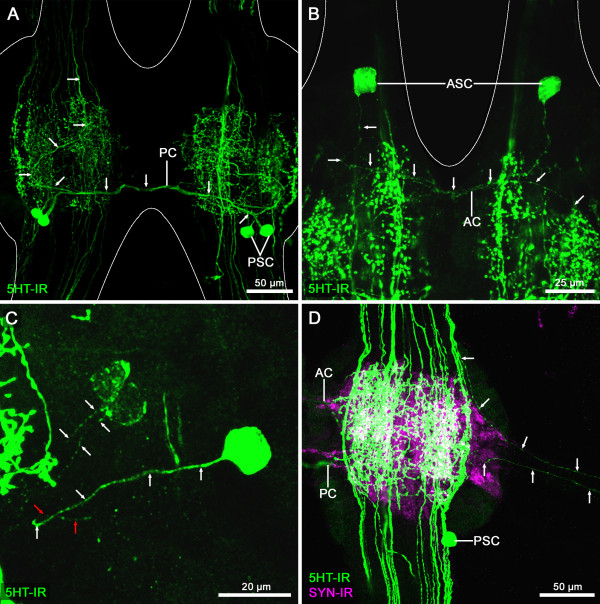
**Morphology of serotonin**-**immunoreactive neurites in the ganglia of the ventral nerve cord.** Confocal laser-scans of trunk ganglia of *Speleonectes tulumensis* (**A, C, D**) and *Godzilliognomus frondosus* (**B**) labeled for serotonin-immunoreactivity (5HT-IR, green) and synapsin (SYN-IR, magenta). (**A**) The axons originating from the posterior 5HT-ir cells (PSC) extend over the posterior commissure (PC) (arrows). In the contralateral hemiganglion, these axons grow in a curve medially and anteriorly into the connective (arrows; shown for *Speleonectes tulumensis*). (**B**) The axons of the anterior 5HT-ir cells (ASC) project posteriorly to the anterior commissure (AC) and grow further posterior into the contralateral neuropil (arrows; shown for *Godzilliognomus frondosus*). (**C**) Neurite projections of the MSCs. One single neurite leaves each medial cell and grows in a direction towards the center of the hemiganglion (arrows). The neurite of the strongly labeled cell seems to loop back, growing laterally (red arrows; shown for *Speleonectes tulumensis*). (**D**) Two 5HT-ir neurites are visible in the anterior segmental nerve (arrows). One is associated with a 5HT-ir longitudinal neurite bundle coming from anterior ganglia, the other one runs into the center of the hemiganglia and could not be followed in more detail (shown for *Speleonectes tulumensis*). Abbreviations: 5HT-IR: serotonin-immunoreactivity; AC: anterior commissure; ASC: anterior 5HT-ir cell; PC: posterior commissure; PSC: posterior 5HT-ir cell; SYN-IR: synapsin-immunoreactivity.

The PSCs were found in each TG of all three species. These neurons normally show a strong immunofluorescence allowing for a detailed description of their projection pattern. In each of the investigated species one primary neurite leaves the soma to project contralaterally (monopolar neurons after the terminology of [33,49]) *via* the PC (exemplarily shown for *Speleonectes tulumensis* in Figure 4A; see also Additional files 1, 2, 3). In the contralateral hemiganglion, the neurites turn anteriorly and enter the CON.

The MSCs appear less constant in soma location and intensity of immunofluorescence labeling. In some preparations, the somata are distributed over a larger area of the ganglion, while in others, the somata are bilaterally symmetrical arranged close to the midline (compare Figures 3B, F). Variations in number and position occurred between different individuals and from ganglion to ganglion within one specimen. However, we found no indication for a particularly high variability in a distinct ganglion. Usually, one MSC shows strong 5HT-IR, whereas the other two are faintly labeled (Figures 3B and 4C, Additional file 2). This is not abnormal because other investigators of 5HT-IR in Euarthropoda have also reported the presence of somata of weaker intensity apart from robustly labeled neurons, which could reliably be detected in all examined preparations (e.g., [44,47,50]). In our preparations, three MSCs could usually be detected in at least one hemiganglion. Based on the bilateral symmetry of the nervous system, we conclude that the presence of three immunofluorescent MSCs in one hemiganglion indicates also the existence of three counterparts in the contralateral hemiganglion. In some cases, less then three MSCs were identified in both hemiganglia of a given segment. However, this phenomenon seems not to be associated with a particular ganglion or region within the trunk (see Table 1). Undetectable neurons might contain rather low concentrations of serotonin close to the immunocytochemical detection threshold. Alternatively, the weak fluorescence might get lost in the background labeling of surrounding neuropil. To provide an estimate of the variability in detectable soma labeling, and to exclude the possibility that certain staining patterns are confined to particular ganglia, we show the number of MSCs in the corresponding left and right halves of the 5^th^, 15^th^, and 25^th^ TGs in *Cryptocorynetes haptodiscus* (Table 1).

**Table 1:** **Number of medial serotonin**-**immunoreactive neurons in selected ganglia of *Cryptocorynetes haptodiscus***

	**Specimen 1**	**Specimen 2**	**Specimen 3**	**Specimen 4**
TG 5	3 / 3	1 / 3	3 / 2	3 / 3
TG 15	3 / 3	2 / 2	3 / 3	1 / 3
TG 25	3 / 3	3 / 3	1 / 3	2 / 1

Displayed is the number of detected medial 5HT-ir neurons (MSCs) in both hemiganglia (left / right) in the 5th, 15th, and 25th trunk ganglia (TG) in the four investigated specimens of *Cryptocorynetes haptodiscus*.

In the three studied species, the somata of the MSCs send one short, unbranched neurite into the neuropil of the hemiganglion (exemplarily shown for *Speleonectes tulumensis* in Figure 4C; Additional file 4), indicating that these neurons are of the monopolar type. We could not resolve whether these neurons project contralaterally or remain on the ispsilateral side. In some cases, we found the medial projecting primary neurite of the intense labeled MSCs looping back laterally (Figure 4C, red arrows), hinting towards an ipsilateral projection pattern.

In the anterior part of each ganglion, another 5HT-ir cell pair could be detected in *Godzilliognomus frondosus* and *Speleonectes tulumensis* (ASC; Figure 3A-C; Additional files 5 and 6). In both species, a primary neurite projects from this pair *via* the AC into the contralateral hemiganglion and then turns posteriorly (exemplarily shown for *Godzilliognomus frondosus* in Figure 4B; Additional files 5 and 6). Due to the intense labeling of the neuropil, the projection could not be traced further.

**Figure 5:**
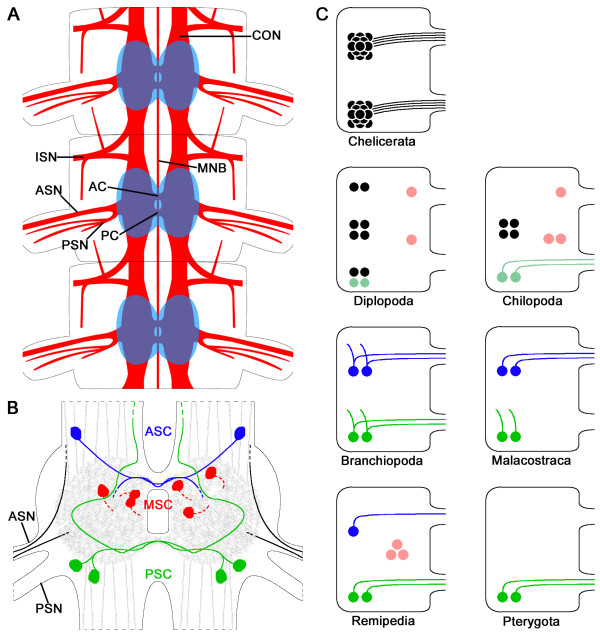
**Neuroanatomy and serotonin**-**immunoreactivity in Remipedia and ground patterns of serotonin**-**immunoreactive neurons in Euarthropoda.** (**A**) The cell cortex is colored in blue, neuronal tissue revealed by acetylated α-tubulin in red. The segmental nerves (ASN and PSN), the intersegmental nerve (ISN), the anterior and posterior commissure (AC and PC), the unpaired longitudinal medial neurite bundle (MNB) and the connectives (CON) are indicated for three trunk segments. (**B**) The 5HT-ir neurons in Remipedia can be distinguished in three groups: one pair of anterior 5HT-ir cells (ASC; blue), three pairs of medial 5HT-ir cells (MSC; red) and two pairs of posterior 5HT-ir cells (PSC; green). The fine homogenous network of 5HT-IR in the neuropil and projections into the CONs are indicated in gray. Two 5HT-ir projections (black) with unknown origin and target region could be observed in the ASN. (**C**) Ground patterns of 5HT-ir neurons in the ventral nerve cord of major euarthropod taxa (modified after [34]). Blue, green and red neurons indicate the anterior, posterior and medial cells, respectively. For cells drawn in unsaturated hues, a potential homology can only be surmised based on soma location. Black neurons represent cells for which no clear homology has been assumed to pancrustacean cells. Abbreviations: AC: anterior commissure; ASC: anterior 5HT-ir cell; ASN: anterior segmental nerve; CON: connective; ISN: intersegmental nerve; MNB: unpaired longitudinal median neurite bundle; MSC: medial 5HT-ir cell; PC: posterior commissure; PSC: posterior 5HT-ir cell; PSN: posterior segmental nerve.

In addition, we found two 5HT-ir neurites in the ASN of each ganglion in the three species (exemplarily shown for *Speleonectes tulumensis* in Figure 4D; Additional files 1 and 7). One of these processes is connected to the most lateral 5HT-ir longitudinal neurite bundle in the anterior ipsilateral CON. The other one is associated with the center of the ipsilateral hemiganglion of the same segment. Generally, the neuropil contains a fine homogenous network of 5HT-ir neurites and varicosities. From the neuropil, intensely labeled neurites extend into the bilateral CONs and are arranged in several parallel longitudinal neurite bundles (Figures 3A and 4D). For all three species, we counted approximately 15 to 20 axons in the CONs. No modifications of the 5HT-ir neurons were observed in ganglia innervating special organs, e.g., in the segments 7 and 14 which host female and male gonopores. The general neuroanatomy of the ventral nerve cord and the distribution and morphology of 5HT-ir neurons are summarized in Figure 5A and B.

## Discussion

### General morphology of the ventral nerve cord

The ventral nerve cord of Remipedia resembles a rope-ladder-like structure (Figures 2A and 5A), most likely a plesiomorphic feature among euarthropods. The MNB has been described in representatives of Malacostraca [51-54] and Mystacocarida [39], but is missing in all studied representatives of Branchiopoda: *Artemia salina*[55], *Leptodora kindtii*[56], *Triops cancriformis*[41], *Leptestheria dahalacensis* and *Cyclestheria hislopi*[42]. A corresponding neurite bundle has also been described in various representatives of Hexapoda [57], but not in Myriapoda [57,58] or Chelicerata [59]. Thus, the MNB might be a plesiomorphy of Pancrustacea. Its absence in Branchiopoda could be due to reduction and hence an apomorphy of this group [39,41].

### Serotonin-immunoreactive neurites in peripheral nerves and connectives

All studies on the serotonergic system in the ventral nerve cord of euarthropods mention 5HT-ir neurites or neurite bundles in the CONs, which travel along the ventral nerve cord interconnecting the segmental ganglia and joining the ventral nerve cord and the brain. There are between 15 to 20 parallel longitudinal 5HT-ir neurites in the CONs of Remipedia (Figures 3A and 4D). It is a common feature in Pancrustacea that 5HT-ir neurons project through several segments in ascending or descending pathways (e.g., [60,61]). Among the many immunofluorescent cell processes, we resolved that the neurites from the PSCs extend anteriorly into the CONs, indicating an ascending pathway. In Decapoda and Syncarida, these neurites are arranged in three distinct tracts called median, central and lateral fiber bundles [60-64]. Furthermore, one single neurite is described in the trunk segments of Mystacocarida [39], four axons are known from Isopoda [37] and approximately 24 axons project in the CONs of the chelicerate *Limulus polyphemus*[65]. Thus, longitudinal 5HT-ir neurites are a common feature among euarthropods, but there are differences in the number and arrangement of 5HT-ir projections between taxa.

Several studies on Euarthropoda describe 5HT-ir neurites within segmental nerves as observed in our study or an immunoreactive plexus surrounding some of the segmental nerves (e.g., Chelicerata: [36,65]; Crustacea: [33,38,40,60-63]; Hexapoda: [44,47]). Because serotonin is released into the haemolymph and acts as a circulating hormone (e.g., [62]) the immunoreactive neurites in the ASN of Remipedia might be projections to peripherally located endocrine release areas as suggested for a copepod [40].

### Comparative neuroanatomy and remipede phylogeny

**Figure 6:**
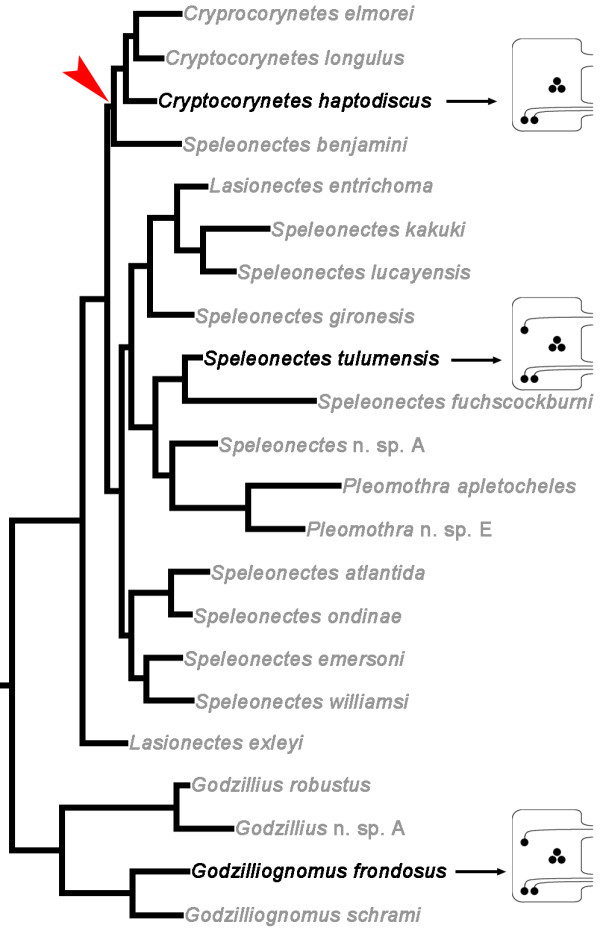
**Distribution of serotonin**-**immunoreactive neurons mapped on the current phylogeny of Remipedia.** 50% majority-rule consensus tree of Remipedia based on a Bayesian analysis of cytochrome oxidase c subunit 1 sequence data (adapted from [66]). The three species that were investigated in this study are written in black. The schematic drawings represent the distribution of 5HT-ir neurons in one hemiganglia of the corresponding species. The loss of the anterior 5HT-ir neuron (ASC) might be an apomorphy of the clade of the genus *Cryptocorynetes* plus *Speleonectes benjamini* (indicated by red arrowhead).

All ganglia of the trunk segments in an individual specimen showed a similar pattern of 5HT-IR. Moreover, this pattern is highly conserved within the three investigated species, indicating serial and interspecific homology for the individually identified serotonin positive neurons. Based on the interspecific comparison, we suggest that the ground pattern of 5HT-ir neurons of Remipedia comprises one anterior, three medial and two posterior pairs of neurons per ganglion (ASC, MSC, and PSC, respectively) (Figure 5B, C). Although the ASC is missing in *Cryptocorynetes haptodiscus*, we included this neuron into the ground pattern, because two species possess this neuron and one of them (*Godzilliognomus frondosus*) is likely a representative of an early lineage within Remipedia [66] (Figure 6).

Although we could only investigate specimens from three species, some general considerations concerning the phylogenetic relationships within Remipedia might be possible. *Speleonectes tulumensis* and *Cryptocorynetes haptodiscus* belong to the Speleonectidae, whereas *Godzilliognomus frondosus* is a representative of the Godzilliidae. However, the phylogenetic relationships within Remipedia are currently being re-evaluated [66]. First results based on an analysis of cytochrome oxidase c subunit 1 sequences from 23 species of Remipedia revealed paraphyletic Godzilliidae and Speleonectidae (Figure 6; compare [66]). In this tree, the basal clade to all other Remipedia is composed of the genera *Godzilliognomus* and *Godzillius*. The three *Cryptocorynetes* species together with *Speleonectes benjamini* are positioned in a more derived clade as a sister group to the remaining species of *Speleonectes*, including *Speleonectes tulumensis*. The status of the genus *Cryptocorynetes* has been already discussed by Koenemann et al., who suggested creating a new taxon comprising the species of this genus [67]. Based on the discussed phylogenetic tree, the loss of the ASC might be an apomorphy of the *Cryptocorynetes* clade (Figure 6).

### Euarthropod phylogeny, comparative neuroanatomy, and neural ontogeny

The distribution and morphology of 5HT-ir neurons have been studied in a variety of euarthropod species, especially crustaceans and hexapods. Similarities in the neuroanatomy of 5HT-ir cells led to the suggestion that some of these neurons may be homologous. In this context, ground patterns for the major taxa have been reconstructed for phylogenetic comparisons (Figure 5C) [33,34,43,68].

According to these studies, the serotonergic system in the ventral nerve cord of Crustacea and Hexapoda can be divided into an anterior and a posterior group, each consisting of at the most two bilateral pairs of neurons. The ground pattern of Branchiopoda and Malacostraca possess the same number of neurons: two anterior and two posterior bilateral pairs. However, there are differences in the morphology of the neurites. Each branchiopod neuron possesses an ipsi- and a contralateral projection (bipolar neurons after the definition of [33,49]), whereas the malacostracan neurons only show one axon with contralateral projections in the anterior group and ipsilateral projections in the posterior group (Figure 5C; compare [68]). In Pterygota however, the anterior pairs of neurons are missing. The remaining posterior cells project one axon each *via* the PC into the contralateral neuropil. According to a preliminary description of Harzsch, two anterior and two posterior 5HT-ir pairs and an additional medial neuron are present in the mesothoracic ganglion of the wingless insect *Thermobia domestica* (Zygentoma) [34,69]. This observation that needs elaboration in additional taxa of wingless insects suggests that the hexapod ground pattern comprises two anterior and two posterior 5HT-ir pairs, as *Thermobia domestica* is the most basal hexapod taxon where 5HT-IR has been studied.

The other major lineages of Euarthropoda, the Myriapoda and Chelicerata, differ markedly in their distribution of 5HT-ir neurons in comparison to Crustacea and Hexapoda [34]. In Chelicerata, 5HT-ir neurons form an anterior and a posterior cluster comprising approximately a dozen neurons with contralateral projections. In Myriapoda, 5HT-ir neurons are arranged in groups of at most four neurons in different positions within the ganglia (Figure 5C). Whether the clusters of 5HT-ir neurons in Chelicerata and Myriapoda correspond to the cell pairs in Crustacea and Hexapoda is unclear (discussed in detail in [34,70]).

Differences in the number, clustering, and distribution pattern might be explained by distinct strategies of neurogenesis among respective taxa. In Chelicerata and Myriapoda, the nervous system is formed from clusters of postmitotic neuronal precursors which migrate from the neuroectoderm, while in Crustacea and Hexapoda individual neural stem cells termed neuroblasts generate defined cell lineages [71-73]. All serotonergic neurons in the ventral nerve cord of two species of locusts, *Schistocerca americana* and *Melanoplus differentialis*, and the fruitfly *Drosophila* are progeny of the neuroblast 7–3 [74-76]. Unfortunately, the lineages of 5HT-ir cells in Crustacea are still unknown. Comparisons between distantly related insect and crustacean embryos revealed a stereotyped pattern of neural precursors and a common plan of neuronal development [77]. This finding initiated a discussion of possible homology among insect and crustacean neuroblasts, despite distinct differences in the segregation pattern from the neuroectoderm [6,29,78-80]. Because of their relative position, proliferation pattern and type of progeny, homology of neuroblasts between insects and malacostracans has been suggested [53,80,81]. Along these lines, we also propose the hypothesis that the posterior immunoreactive neurons are homologous in Remipedia, Hexapoda and other Crustacea. Similar evidence indicates homology of the anterior 5HT-ir neurons between Remipedia and other Crustacea. This type of neuron appears to be absent in the ground pattern of Pterygota (Figure 5C).

Harzsch and co-workers suggested that differences in the mode of neural ontogeny account for the differences in number of 5HT-ir neurons [70]. As Chelicerata possess clusters of neuronal precursors, it might be reasonable that clusters of 5HT-ir neurons and not single cells result from this mode of neurogenesis. In contrast, the asymmetrically dividing neuroblasts in Crustacea and Hexapoda generate specific lineages that comprise small numbers of individual 5HT-ir neurons.

Following the generally accepted opinion that Chelicerata represents the plesiomorphic mode of neurogenesis, whereas the Hexapoda and Malacostraca represent a rather derived mode, the serotonergic systems of the ventral nerve cord seem to have undergone a simplification process. A comparative analysis of the serotonergic neurons of crustacean and hexapod taxa indicates that the ground pattern of Branchiopoda represents the complete serotonergic system, which is then reduced in more derived taxa from four pairs with bipolar projections in Branchiopoda to four pairs with only monopolar projections in Malacostraca. This loss of bipolar neurons points towards a close relationship of Malacostraca, Remipedia and Hexapoda, all of which possess only monopolar neurons. Remipedia and Hexapoda show only contralateral projections, whereas the posterior neurons of Malacostraca project ipsilaterally.

Interesting is that in almost all molecular studies including recent phylogenomic data, Branchiopoda are inferred as closer related to Remipedia and Hexapoda than Malacostraca (e.g., [8,9,28]). However, strong evidence from morphological studies (e.g., [14,15,22,82]) supports an ambiguous, equally likely evolutionary scenario, in which Malacostraca, Remipedia and Hexapoda are closely related. Our study presents new characters that potentially could resolve these contradicting results if extended to more taxa. In this context, the Cephalocarida play an important role, because they were recovered in two recent molecular studies as closest relatives to Remipedia [8,19]. Based on long branch effects and inhomogeneous substitution rates, this grouping was critically discussed since its first molecular reconstruction [23]. A sister group relationship is also not supported by recent anatomical studies of the brain of the cephalocarid *Hutchinsoniella macracantha*[83].

In addition to the ASCs and PSCs, we detected three MSCs in Remipedia. It will be interesting to find corresponding cells in other euarthropod taxa. One medial neuron has been described in the mesothoracic ganglion of the silverfish *Thermobia domestica*[34,69] and medial 5HT-ir neurons have also been assigned to the ground plan of Myriapoda [34]. To facilitate meaningful comparisons, it will be necessary to resolve their detailed projection pattern in different euarthropod taxa.

Previous neuroanatomical studies on the remipede brain [14,15,22] discovered several synapomorphies with Malacostraca and Hexapoda, such as the layout of the olfactory system including its projection neuron pathway to the protocerebrum and the architecture of the central complex. Here, we resolved the rather simple organized serotonergic system of the homonomously segmented ventral nerve cord indicating that the remipede nervous system comprises a mixture of ancestral and derived anatomical features. However, it is in line with previous neuroanatomical studies [14,15,22] in support of a polytomic clade of Malacostraca, Remipedia and Hexapoda.

## Conclusions

Remipedia possess a rope-ladder-like arrangement of segmental ganglia, a feature considered plesiomorph for Euarthropoda. The neuroanatomical description of the 5HT-ir cells in the ventral nerve cord of Remipedia supports the proposed homology of these identifiable neurons in Pancrustacea. Due to position and projection pattern, we assume that the anterior and posterior 5HT-ir neurons are homologous between Remipedia, other Crustacea and Hexapoda. Comparing the ground patterns from pancrustacean taxa reveals similarities between Remipedia and Hexapoda. Our findings on the ventral nerve cord support the opinion that Remipedia are a more derived crustacean lineage and do not contradict the phylogenetic investigations that propose a sister group relationship of Remipedia and Hexapoda [9,20,82], although the architecture of the remipede brain shows slightly more resemblance to that of malacostracan crustaceans [14,15,22]. The relevance of the medial 5HT-ir neurons in the central nerve cord of Remipedia and Zygentoma for phylogenetic analyses has to be addressed in the future. To answer if these neurons can be homologized and if this feature is another argument for a sister group relationship of Remipedia and Hexapoda needs to be investigated in further studies.

## Methods

### Collection and tissue processing

Four individuals of *Cryptocorynetes haptodiscus* and one of *Godzilliognomus frondosus* were obtained from Sawmill Sink on Abaco Island, Bahamas. Two specimens of *Speleonectes tulumensis* came from the Cenote Crustacea on the Yucatan Peninsula, Mexico. Directly after collection, each specimen of *Speleonectes tulumensis* was cut into three parts in order to assure the penetration of the tissue by the fixative. All other specimens were fixed without any dissection. Animals were fixed in 4% paraformaldehyde dissolved in phosphate-buffered saline (PBS, 10 mM sodium phosphate, 150 mM sodium chloride, pH 7.4) for up to five days. The tissue was washed four times in PBS for at least 30 min each and stored at 4°C in PBS with 0.5% sodium azide until use.

After short incubation with Poly-L-Lysin, the tissue was embedded in 4% agarose dissolved in aqua dest. at approximately 50°C. After cooling to room temperature, the blocks were trimmed and sectioned horizontally in 50–60 μm using a vibratome (Hyrax V 50, Zeiss).

### Immunocytochemistry

All steps of immunocytochemistry were performed on a shaker with smooth agitation at room temperature. All washing steps were conducted with 0.2% Triton X-100 (PBS-TX 0.2%) for at least 15 min if not stated differently. Horizontal sections were permeabilized for 45 min with 0.3% Saponin dissolved in PBS-TX 0.2%. After three washing steps, nonspecific binding of antibodies was blocked by incubating in 5% normal goat serum (Vector) in PBS-TX 0.2% for 3 h. Subsequently, sections were incubated for 48 hours at 4°C with blocking solution containing the following antibodies: the polyclonal antibody rabbit anti-serotonin (Sigma; dilution 1:2000), the monoclonal antibodies mouse anti-acetylated α-tubulin (Sigma; 1:500) and mouse anti-synapsin “SYNORF1” (DSHB University of Iowa; 1:30) [84]. Three washing steps were followed by incubation with a mixture of the secondary antibodies goat anti-rabbit Cy3-conjugated (Jackson Immuno Research Laboratories; 1:250) and goat anti-mouse Alexa Fluor 488-conjugated (Molecular Probes; 1:250) plus 4′6-diamidine-2-phenylidole-dihydrochloride (DAPI) for counterstaining the nuclei (1 μg/ml) in blocking solution overnight at 4°C. Sections were washed again three times and an additional time in PBS and mounted on glass slides in Mowiol.

### Antibody specificity

In this study, we used a polyclonal rabbit antiserum raised against a serotonin creatinine sulfate complex conjugated to bovine serum albumin as the immunogen (Sigma, cat. no. S5545, lot no. 108K4868), a monoclonal mouse anti-*Drosophila* synapsin antibody (“SYNORF1”, Developmental Studies Hybridoma Bank, University of Iowa, 1:30) raised against a *Drosophila* GST-synapsin fusion protein and a monoclonal mouse anti-acetylated α-tubulin (Sigma, cat. no. T6793, lot no. 059K4823, clone 6-11B-1) raised against acetylated tubulin of *Strongylocentrotus purpuratus* (sea urchin). Because of the difficulty of collecting specimens of Remipedia, only a low number of specimens were available. Thus, no experiments concerning the specificity of antisera could be conducted. However, 5HT-IR was investigated in a variety of invertebrate phyla [48], including studies on all kinds of Euarthropoda (Chelicerata and Myriapoda: e.g., [34,36]; Hexapoda: e.g., [85-88]; Crustacea: e.g., [22,33,39,41,83]). Several of these studies used the same antiserum as in this account [22,41,88]. This let suggest a highly conserved antigen and a specific labeling of serotonin.

The antisera used for acetylated α-tubulin labeling is a monoclonal antibody raised against acetylated α-tubulin from the sea urchin *Strongylocentrotus purpuratus* (Sigma, cat. no. T6793, lot no. 059K4823, clone 6-11B-1). This antibody reacts with acetylated α-tubulin over a wide range of species such as plant, human, pig, monkey, invertebrates, hamster, bovine, chicken, rat, frog, protista and mouse (see datasheet manufacturer) and was utilized in numerous studies on the nervous system of the major crustacean taxa (e.g., Branchiopoda: [41]; Cephalocarida: [83]; Malacostraca: [52]; Maxillopoda: [39]; Remipedia: [22]).

The used synapsin antiserum is a monoclonal mouse anti-*Drosophila* synapsin antibody (“SYNORF1”, Developmental Studies Hybridoma Bank, University of Iowa) raised against a *Drosophila* GST-synapsin fusion protein. This antibody stained neuropil structures over a wide range of euarthropod taxa, for example Crustacea (Branchiopoda: [55,89]; Malacostraca: [52,90,91]; Remipedia: [22]), Hexapoda (e.g., [92,93]), Chilopoda [94] and the spider *Cupiennius*[95]. Additionally, in western blots of brain tissues of *Drosophila* and the crustacean *Coenobita* (Anomura) identical bands were stained by the synapsin antibody, which suggests that the epitope for SYNORF 1 is strongly conserved between *Drosophila* and *Coenobita*[91]. These results and the similar staining pattern of synaptic neuropils in different euarthropod taxa lead to the suggestion that the synapsin antibody reacts with a highly conserved epitope.

The three antibodies stain similar structures in a variety of euarthropod species and were used in a previous study of the nervous system of Remipedia [22]. Thus, we suggest that these antisera in fact label specific structures in the remipede ventral nerve cord.

### Microscopy and image acquisition

Confocal images and z-stacks were taken with a Leica TCS SP5 confocal laser-scanning microscope using Leica LAS AF software. Z-series processing including maximum projections of confocal stacks, contrast and brightness enhancement, and movie preparation was conducted with NIH ImageJ, v. 1.46r (Rasband, W.S., ImageJ, U.S. National Institutes of Health, Bethesda, MD, http://rsb.info.nih.gov/ij/). Photographs were arranged using Adobe Photoshop 6.0 (San Jose, CA).

### Animal ethics

Texas A&M University is an AAALAC, international accredited facility which conducts all vertebrate animal activities involving research, teaching, and testing according to the regulations set forth by the Animal Welfare Act, the Guide for the Care and Use of Laboratory Animals, and the Public Health Service Policy. The work conducted by Dr. Iliffe and colleagues involved the use of invertebrates which are not specifically covered by the regulations previously listed, but are also afforded the highest standard of care when used in research, teaching, or testing. No work on living specimens was conducted in Europe. All procedures in this investigation complied with international and institutional guidelines, including the guidelines for animal welfare as laid down by the German Research Council (DFG).

## Abbreviations

5HT-IR: Serotonin-immunoreactivity; 5HT-ir: Serotonin-immunoreactive; AC: Anterior commissure; ASC: Anterior serotonin-immunoreactive cell; ASN: Anterior segmental nerve; CON: Connective; DAPI: 4′6-diamidine-2-phenylidole-dihydrochloride; ISN: Intersegmental nerve; MNB: Unpaired longitudinal median neurite bundle; MSC: Medial serotonin-immunoreactive cell; NUC: Nuclear marker; Numbers 1–3: Neurite bundles originating from the posterior segmental nerve; PBS: Phosphate-buffered saline; PBS-TX: 0.2% Phosphate-buffered saline containing 0.2% Triton X-100; PC: Posterior commissure; PSC: Posterior serotonin-immunoreactive cell; PSN: Posterior segmental nerve; SYN-IR: Synapsin-immunoreactivity; TG: Trunk ganglion; TUB-IR: Acetylated α-tubulin-immunoreactivity.

## Competing interests

The authors declare that they have no competing interests.

## Authors’ contributions

TMI and BMvR conducted the sampling and fixation of specimens. TS carried out the vibratome sectioning, the immunocytochemical experiments and the confocal laser-scanning microscopy. TS drafted the first version of the manuscript and all other authors assisted in drafting the manuscript. All authors read and approved the final manuscript.

## Supplementary Material

Additional file 1 **Projections of posterior 5HT-ir neurons in *Speleonectes tulumensis.*** The movie consists of a z-stack showing the contralateral projections (arrows) of the posterior 5HT-ir neurons (PSC) in *Speleonectes tulumensis via* the posterior commissure. The maximum projection of this z-stack is shown in Figure 4A. This projection corresponds to the situation in *Godzilliognomus frondosus* (Additional file 2) and *Cryptocorynetes haptodiscus* (Additional file 3). An image has been scanned each 2.5 μm covering a z-distance of 22.5 μm (in total 10 images). Abbreviations: PSC: posterior 5HT-ir cell. Click here for file

Additional file 2 **Distribution and projections of 5HT-ir neurons in *Godzilliognomus frondosus.*** The movie consists of a z-stack showing the distribution of anterior, medial and posterior 5HT-ir neurons (ASC, MSC, and PSC, respectively). The contralateral projections (arrows) of the posterior 5HT-ir neurons (PSC), the two 5HT-ir neurites in the anterior segmental nerve (arrowheads), and short neurite projection of a MSC (double arrowhead) are indicated. The maximum projection of this z-stack is shown in Figure 3A. An image has been scanned each 2.5 μm covering a z-distance of 47.5 μm (in total 20 images). Abbreviations: ASC: anterior 5HT-ir cell; MSC: medial 5HT-ir cell; PSC: posterior 5HT-ir cell. Click here for file

Additional file 3 **Projections of posterior 5HT-ir neurons in *Cryptocorynetes haptodiscus.***The movie consists of a z-stack showing the contralateral projections (arrows) of the posterior 5HT-ir neurons (PSC) in *Cryptocorynetes haptodiscus via* the posterior commissure. This projection corresponds to the situation in *Speleonectes tulumensis* (Figure 4A, Additional file 1) and *Godzilliognomus frondosus* (Additional file 2). An image has been scanned each 1.0 μm covering a z-distance of 24 μm (in total 25 images). Abbreviations: MSC: medial 5HT-ir cell; PSC: posterior 5HT-ir cell. Click here for file

Additional file 4 **Projections of medial 5HT-ir neurons in *Speleonectes tulumensis.***The movie consists of a z-stack showing the projections of the medial 5HT-ir neurons (MSC) in the right hemiganglion of *Speleonectes tulumensis* (arrows). The maximum projection of this z-stack is shown in Figure 4C. An image has been scanned each 0.5 μm covering a z-distance of 9 μm (in total 19 images). Abbreviations: MSC: medial 5HT-ir cell. Click here for file

Additional file 5 **Projections of anterior 5HT-ir neurons in *Godzilliognomus frondosus.***The movie consists of a z-stack showing the contralateral projections (arrows) of the anterior 5HT-ir neurons (ASC) in *Godzilliognomus frondosus via* the anterior commissure. The maximum projection of this z-stack is shown in Figure 4B. This projection corresponds to the situation in *Speleonectes tulumensis* (Additional file 6). An image has been scanned each 1.5 μm covering a z-distance of 13.5 μm (in total 10 images). Abbreviations: ASC: anterior 5HT-ir cell. Click here for file

Additional file 6 **Projections of the anterior 5HT-ir neurons in *Speleonectes tulumensis.***The movie consists of a z-stack showing the projections of the anterior 5HT-ir neurons (ASC) in *Speleonectes tulumensis* (arrows). This projection corresponds to the situation in *Godzilliognomus frondosus* (Figure 4B, Additional file 5). An image has been scanned each 1.0 μm covering a z-distance of 21 μm (in total 22 images). Abbreviations: ASC: anterior 5HT-ir cell. Click here for file

Additional file 7 **5HT-ir neurites in the anterior segmental nerve in *Speleonectes tulumensis.***The movie shows the two 5HT-ir neurites in the anterior segmental nerve of the ganglia in *Speleonectes tulumensis*, which have been described in Figure 4D as maximum projection. An image has been scanned each 0.5 μm covering a z-distance of 12 μm (in total 25 images). Click here for file
